# Teratoma during Pregnancy with Positive Estrogen and Progesterone Receptors and Elevated Ca19-9 Antigen Levels

**DOI:** 10.1155/2012/970845

**Published:** 2012-10-08

**Authors:** Nilgun Kanlioglu Kuman, Salih Cokpinar, Ertan Yaman, Ibrahim Meteoglu, Fisun Karadag

**Affiliations:** ^1^Department of Thoracic Surgery, Faculty of Medicine, Adnan Menderes University, 09000 Aydin, Turkey; ^2^Department of Pathology, Faculty of Medicine, Adnan Menderes University, 09000 Aydin, Turkey; ^3^Department of Pulmology, Faculty of Medicine, Adnan Menderes University, 09000 Aydin, Turkey

## Abstract

We present a 27-year-old female patient admitted with an anterior mediastinal mass. She complained of chest discomfort and hemoptysis which began seven months prior. She had given birth five months prior. Thoracic X-ray showed an anterior mediastinal mass. Thorax computed tomography (CT) confirmed a well-defined anterior mediastinal mass with 13 × 12 cm diameter, extending to the right hemithorax. It was composed of cystic spaces and discrete areas like soft tissue and fat. Serum Ca 19-9 level was elevated. CT features were consistent with a mature teratoma. During median sternotomy, the tumor revealed adhesions to the right lung and the right subclavian artery. Histologically, the tumor was diagnosed as a mature teratoma. Estrogen and progesterone receptors were detected to be positive in the resected tissue. We conclude that alterations in hormone levels during pregnancy might be the cause of rapid tumor growth which leads to hemoptysis.

## 1. Introduction

Earlydiagnosisof mediastinaltumorsis raresince symptoms usually arise after compression of the theadjacent structures [1]. Most ofthe mediastinaltumorsare asymptomatic and53%are detectedon chest radiographytaken for other reasons [2].

## 2. Case

**Figure 1:**
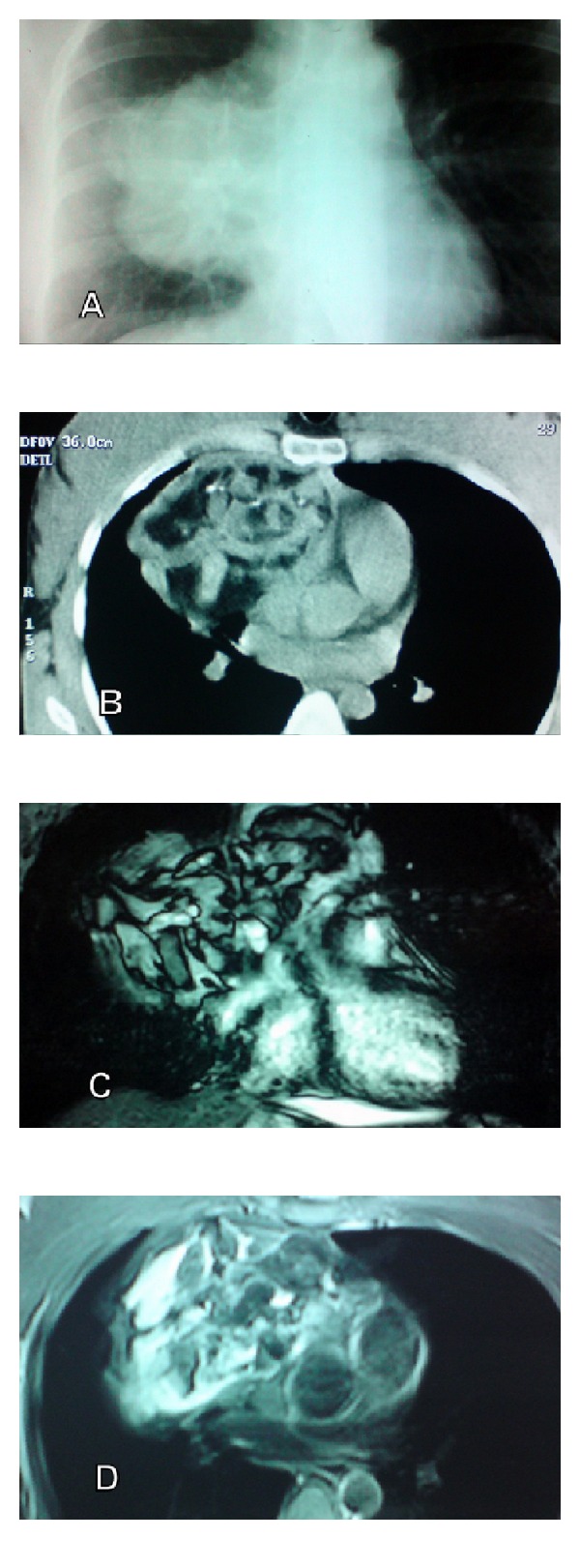
(A) Mediastinal lesion on chest radiography.(B) A mass with heterogenousdensity andcalcificationsin anterior mediastinum onthorax CT.(C) and (D), heart and main vascular structures are displaced by the mass on thoracic MRI.

A 27-year-old female patient had beeninvestigated seven months ago because ofhemoptysis andchest painduringpregnancy. Thechestradiography revealed a 13 × 12 cm hypodense lesion with smooth margins at mediastinum.There was no specific feature in her medical history except a full term birth five months ago.Routine laboratory tests and HCG, AFP, CEA, CA 15-3, and prolactin levels were normal whereas CA 19-9 was elevated (126 U/mL).CT showed a lobulated lesion filling the anterior mediastinum. Lesion contained a large amount of fat, solid components, and calcifications. The mass extended from rightbrachiocephalicvein to therightatrioventricularsulcus and was locatedadjacent tothe branchesof the aortaandpulmonary veins. There were intraparenchymal small cystic areas and a linear density in the anterior segment of right upper lobe. Thoracicmagnetic resonance imaging(MRI) showed amasswith fat, liquid, and solid components, pressing on the heart (Figure 1).

Median sternotomy was performed and a mass lesion with tight adhesions to pericardium, left and right mediastinal pleura, right upper lobe, and brachiocephalic vein was detected in anterior mediastinum. Tumor excision and the right upper lobe wedge resection were performed. During excision, hair, and cartilage tissue protruding from the mass were observed. 

**Figure 2:**
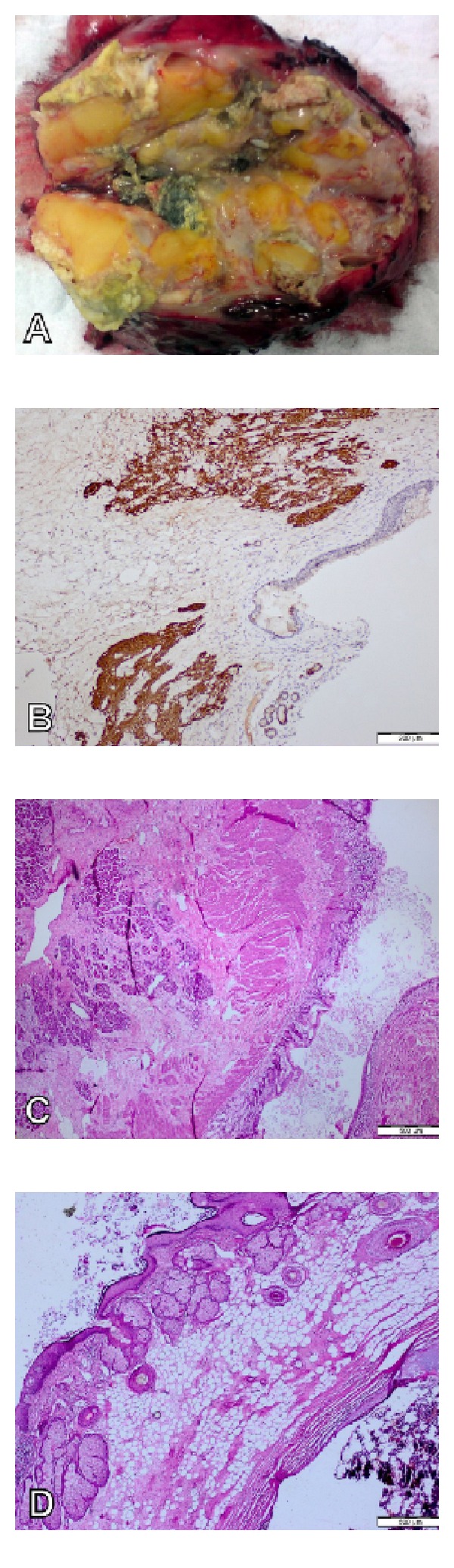
(A) Cystic spaces, fat, cartilage, and hair can be seen insurgical resection material. (B) Glial tissue was stained with GFAP in immunohistochemical study (×40). (C) Muscle,connective tissue,columnar epithelium, and pancreatic tissuewere seen bymicroscopy (HE, ×40).(D) Epidermis,skin appendages, fattissue, and bonetissueareas were seen on microscopic study (HE, ×100).

Pathological specimen was443 grams and 13 × 12 × 6 cm in size. Skin, skin appendages, cartilage, bone tissue, bone marrow elements, respiratory, and gastrointestinal epithelium were observed in microscopic examination. Tissue had mature components, which were stained with glial S100 and GFAP, consistent with teratoma. Emphysematous changes, thickening of alveolar septa, and interstitial mononuclear infiltration were detected in the lung specimen, and the surgical margins were tumor free (Figure 2).

**Figure 3:**
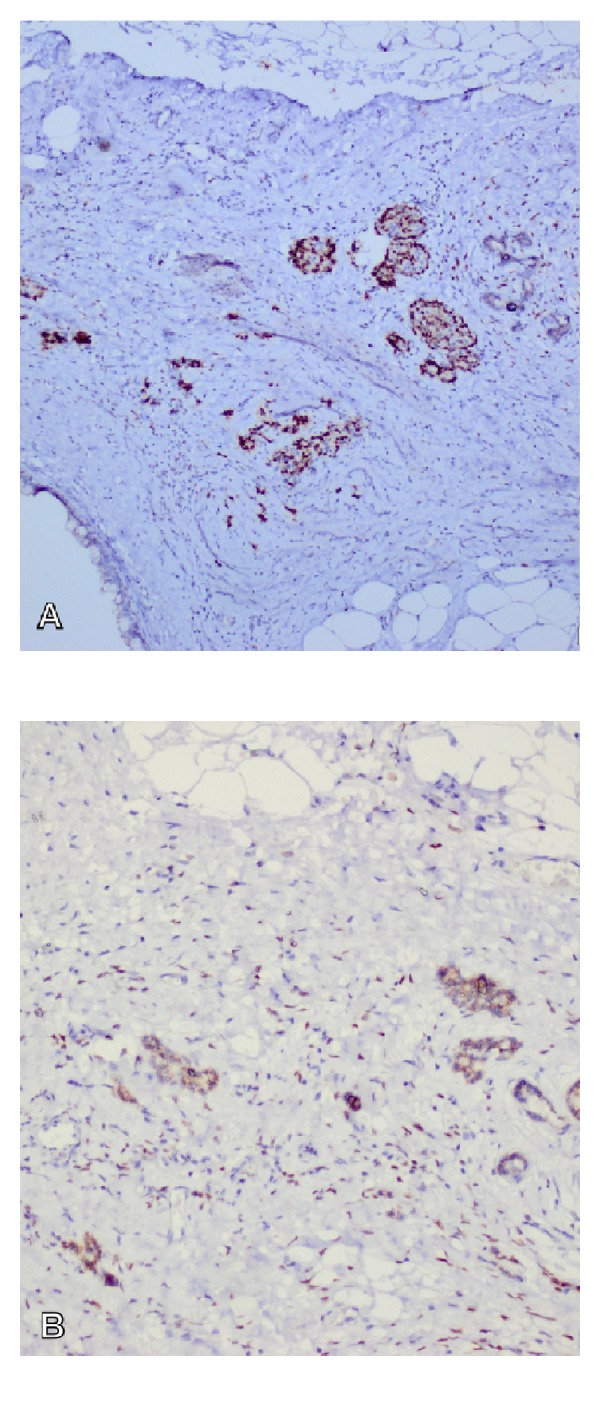
(A)Immunohistochemical PR-positiveglandular structuresand (B) immunohistochemical ER-positiveglandular structureswere seen on image (×100).

Estrogenandprogesteronereceptorswere detected to be positive in the resected tissue (Figure 3).

The patient was discharged on postoperative seventh day.There were no complications during 5 months of followup.

## 3. Discussion

Teratomas were described first in 1953 by Willis as a tumor that contained tissues different from the affected organ [3].95% ofteratomas are placed inthe anterior mediastinum and detected in second frequency after thymomas among the tumors of anterior mediastinum [1, 3]. Teratomas arise from multipotent primitive embryonic cells and mediastinal teratomas may develop from the surrounding cells of third bronchial cleft [3]. Most teratomas contain mesodermal, endodermal and especially mature ectodermal components [1]. Teratomas can produce hormones because they contain some different cell types [4]. Estrogen receptorsandprogesteronereceptorsmay be elevated in tissue like our case. 

Although most teratomas are asymptomatic, symptoms such as chest pain, dyspnea, and cough may be seen. There is no other case in the literature who became symptomatic during pregnancy and treated afterpregnancy like our patient literature. Uyama et al. reported that the alterations in sex hormones in menarche may have played a role in the rapid tumor growth [5]. When teratomas rupture to lung or other adjacent organs, they are always symptomatic. Symptoms have been reported such as hemoptysis, expectoration of hair, and other tissues, pneumonia, acute respiratory distress, pleural empyema. Cardiac tamponade may develop when it ruptured to pericardium [1–3].The etiology of the perforation is controversial. Ischemia, infection, and inflammation were claimed to be the reasons.In addition, cystic teratomas contain pancreatic or salivary gland tissue. The secreted proteolytic or digestive enzymes may cause adhesion or erosion of the surrounding structures. Rupture or fistula formation may develop as a result of chronic inflammation of the cyst wall [2].We did not detect complete rupture of the teratoma in our case; a small amount of hemoptysis occurred possibly due to chronic changes as a result of compression of the lungs. However, rupture of teratoma to the lung should be considered in patients presenting with hemoptysis.Although pressure on the heart and great vessels is infrequent, cardiac symptoms such as atrial fibrillation are reported in the literature [6].

Teratomas are seen in anterior mediastinum with about 8–10 cm size, appearing to protrude into a hemithorax on plain chest radiography [3]. CT has diagnostic value for teratomas because of pathognomonic findings of fat and calcific densities [3]. The tumor invasion into surrounding tissues and structural properties can be evaluated with CT [1].According to CT findings, soft tissue was found in almost all teratomas, while 88% liquid, 76% fat, and 53% calcification were found. MRI is a valuable technique used in determining the relationship with anatomical structures such as mediastinal and hilar vessels and airways [6]. CT and MRI evaluation will be helpful to determine the internal structure of the tumor, the relationship with the surrounding tissue and planning the operation as in our case. Preoperativeserumalphafetoprotein (AFP) andB-HCG levels should be evaluated in suspectedgerm celltumors [6]. In our case, serum HCG, AFP, CEA, CA 15-3, and prolactin were detected at normal levels but CA 19-9 level was increased. Immunohistochemical studies revealed that epithelialtissues containing squamous epithelium or bronchial components have CA 19-9 [7].

Mature teratomas are treated by surgical resection. Sternotomy is the most preferred method to achieve optimal exposure [1, 6, 8].Thoracotomy can be the choice when the tumor is localized to one hemithorax [3]. Teratomas have good prognosis and there is no need for adjuvant chemo- or radiation therapy [8]. According to literature, local recurrence rate is low and the total excision is not necessary if dense adhesions were observed to pericardium, lung, or main vascular structures [3].However, complete resection will enable the correct diagnosis and treatment.

We conclude that alterations in hormone levels during pregnancy such as estrogen and progesterone might have stimulated the sensitive tumorcellsleading totumorgrowth in our case. Thus, the tumor became symptomaticdue to mass effectand increased inflammation led to adhesions tosurrounding tissues.
